# Effects of monosaccharide composition on quantitative analysis of total sugar content by phenol-sulfuric acid method

**DOI:** 10.3389/fnut.2022.963318

**Published:** 2022-08-02

**Authors:** Fangfang Yue, Jinrui Zhang, Jiaxin Xu, Tengfei Niu, Xin Lü, Manshun Liu

**Affiliations:** ^1^College of Food Science and Engineering, Northwest A&F University, Yangling, China; ^2^College of Enology, Northwest A&F University, Yangling, China

**Keywords:** phenol-sulfuric acid method, total sugar content, polysaccharides, monosaccharides, color-rendering capabilities

## Abstract

Phenol-sulfuric acid method is one of the most common methods applied to the analysis of total sugar content during polysaccharides study. However, it was found that the results obtained from the phenol-sulfuric acid method was generally lower than the real total sugar content, especially when acidic monosaccharides were contained in the polysaccharides samples. Therefore, the present study focused to unveil the proposed problem. Based on the optimization of colorimetric conditions, such as optimal wave length of absorption, linearity range, color reaction time and temperature, it indicated that the phenol-sulfuric acid method was a convenient and accurate way for the total sugar content analysis. In addition, the color-rendering capabilities of 10 common monosaccharides were systematically analyzed to obtain a relative correction factor for each monosaccharide relative to glucose, which was proved to be the main reason for the deviation in the detection of total sugar content. Moreover, the key points during the application of phenol-sulfuric acid method were suggested. This study provides a scientific theoretical basis and a reliable experimental research method for the accurate determination of total sugar content by the phenol-sulfuric acid method, and which will also promote the application of this convenient method in the polysaccharides study.

## Introduction

In recent years, polysaccharide, as an important natural macromolecule, have been found to have various functional activities, such as antioxidant (1, 2), immune regulation (3), anti-tumor (4, 5), alleviation of obesity (6) and so on. Although the research biological activities of polysaccharides have been widely studied (7), the detection methods of the physicochemical properties of polysaccharides still need to be further optimized and perfected. The total sugar content is one of the important indicators of the physicochemical characteristics of polysaccharides.

In the industrial production process of polysaccharides, it is often necessary to measure and monitor the content of polysaccharides (8). Due to the wide variety of polysaccharides, the content and existing forms of polysaccharides are also variable (9, 10). Therefore, choosing an appropriate detection method is crucial. The total sugar represents the sum of reducing sugars (glucose, fructose, lactose, etc.) and oligosaccharides (sucrose, etc.) that can be hydrolyzed into reducing sugars under the measurement conditions (11, 12). The determination methods of total sugar content mainly include phenol-sulfuric acid method (2), anthrone-sulfuric acid method (13), full-wavelength enzyme labeling method (14), and direct titration method (15). In the current research, due to the simple detection method, good stability and high sensitivity, phenol-sulfuric acid method is widely used (16). In addition, phenol-sulfuric acid method can also measure methylated sugars, pentoses and polysaccharides, and is not affected by proteins (17, 18).

**Figure 1:**
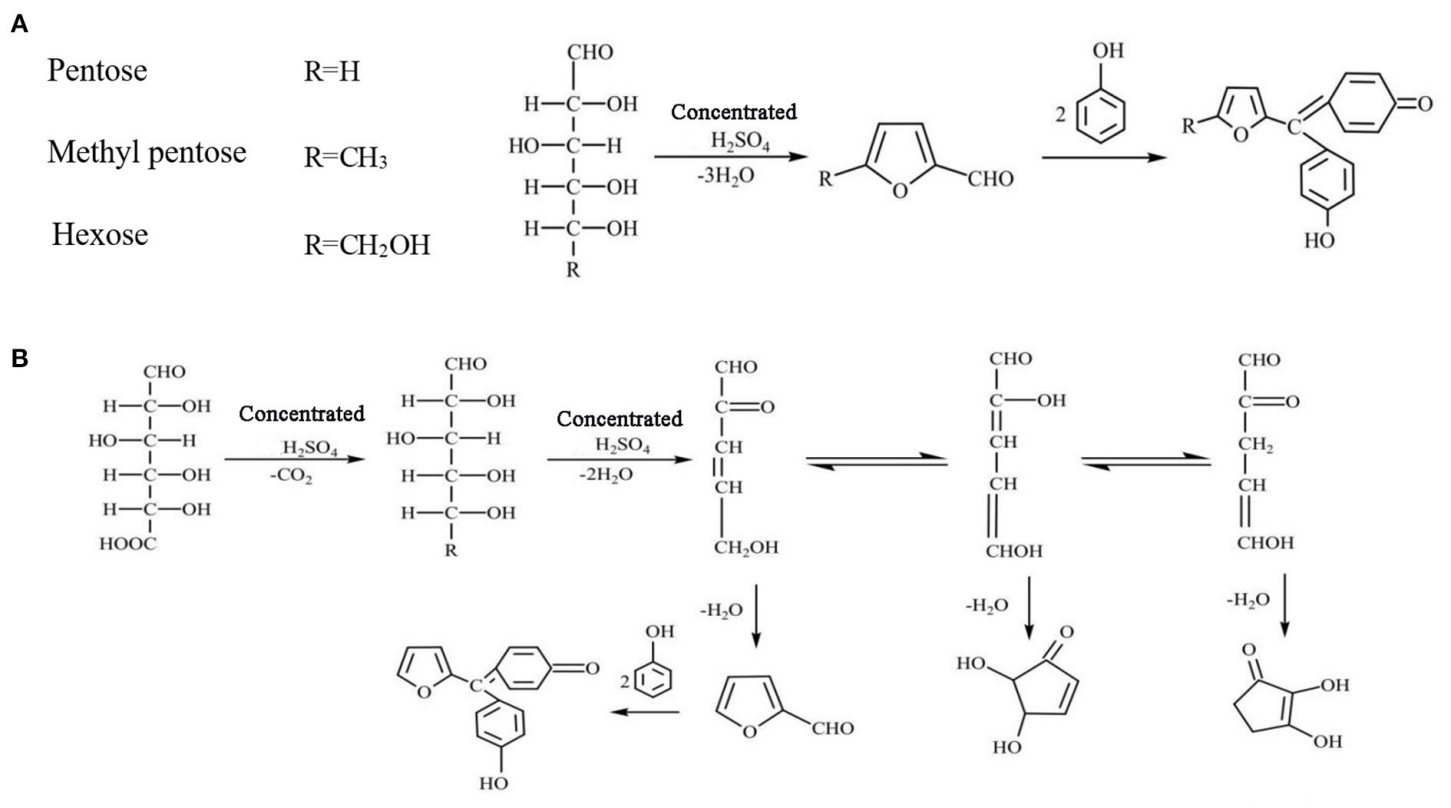
Principle of phenol-sulfuric acid method. **(A)** Pentose, methyl pentose and hexose reaction principle. **(B)** Uronic acid reaction principle.

The determination principle of the phenol-sulfuric acid method is that concentrated sulfuric acid dehydrates polysaccharides to form uronic acid and hydroxyurea formaldehyde, and then condenses with phenol to form orange-red compounds (Figure 1). Within a certain concentration range, the color depth is proportional to the sugar content, which can be determined at OD 490 nm wavelength. However, experimental conditions, such as color development wavelength, color development time, color development temperature, and detection range play an important role in the accuracy of the determination of total sugar content (19). In addition, the calculation of the specific total sugar content needs to be obtained according to the standard curve drawn by standard monosaccharides. At present, glucose is often used as a standard in experiments, which leads to large errors in the determination of sugar content, especially for heteropolysaccharides. Apart from glucose, the hydrolysis of heteropolysaccharides may also generate other monosaccharides, and different monosaccharides have significant differences in structure and physicochemical properties. For example, in terms of functional groups, glucose is aldohexose and fructose is ketohexose (20). Also, ribose, xylose, and arabinose are pentose. And glucose, mannose, galactose, and fructose are hexose (21). In addition, in the process of phenol-sulfuric acid method, pentoses are more likely to react than hexose, the reaction rate of uronic acid is relatively slow, and uronic acid cannot be completely converted. Therefore, it is speculated that there may be significant differences in the color development ability of different monosaccharides after condensation with phenol. Although a great deal of research has been done to improve the phenol-sulfuric acid method, little attention has been paid to the errors caused by standard monosaccharides.

Therefore, this study explored the effect of different monosaccharide compositions on the quantitative analysis of total sugars. Through the independent quantitative analysis of a variety of different monosaccharides, a complete set of color development system for quantitative analysis of total sugars in phenol sulfate was established. The method is conducive to more accurate determination of the total sugar content contained in the present product, and makes up for the defect that the total sugar yield deviates from the actual content due to the large difference in color development of different types of monosaccharides. This experiment improves the rigor and accuracy of total sugar quantitative analysis, and provides a scientific theoretical basis and reliable experimental research methods for future applications in chemical industry, molecular detection and other fields.

## Materials and methods

### Materials and reagents

Glucose (Glu), Fructose (Fru), Mannose (Man), Galactose (Gal), Xylose (Xyl), Arabinose (Ara), Rhamnose (Rha), Fucose (Fuc), Galactose Uronic acid (GalUA), glucuronic acid (GlcUA), purity ≥ 98%, β-dextran (purity ≥ 80%), fructo oligosaccharide (purity ≥ 95%), xylan (purity ≥ 98%) and polygalacturonic acid (purity ≥ 90%) were purchased from Yuanye Biotechnology Co., Ltd. D-trehalose (purity ≥ 99%) was purchased from Shanghai McLean Biochemical Technology Co., Ltd. All of other chemicals and reagents were analytical grade.

### Moisture content detection of monosaccharide standards

Quickly weigh a certain specification of standard monosaccharide. Then, it was dried in an oven with a temperature of 80°C and standard atmospheric pressure for 12 h, and the quality of the standard monosaccharide after drying in the oven was recorded. Calculate the moisture content of the standard monosaccharide by making the difference twice. If the difference between the two weighings does not exceed 0.002 g, it is recorded as constant weight.

### Optimization of the phenol-sulfuric acid method

Using glucose as the standard solution, the detection conditions of the phenol-sulfuric acid method for the determination of total sugar were preliminarily determined by optimizing the color wavelength (300–700 nm), color stability (color stability of 10, 40, 80 μg/mL standard grape solution during the detection process), detection range (0–1,000 μg/mL standard grape solution), and heating conditions at 100°C or room temperature (RT).

### Monosaccharide ultraviolet absorption spectroscopic analysis

First accurately prepare 100 μg/mL standard solutions of 10 kinds of monosaccharides, then accurately draw 0.8 mL of each monosaccharide standard solution, add distilled water to make up to 2.0 mL, and prepare a monosaccharide standard solution with a solution concentration of 40 μg/mL. Then accurately prepare 80% phenol-ethanol solution and store it in a brown bottle at 4°C away from light. Accurately draw 7.5 mL of 80% phenol solution and dissolve it in 96 mL of absolute ethanol to prepare a 6% phenol solution.

Add 1.0 mL of 6% phenol solution (currently prepared) to the working solutions of 10 monosaccharides, respectively, then quickly add 5.0 mL of concentrated sulfuric acid, mix well in time, and stand at RT for 20 min. Distilled water was used as blank control. Use Ultraviolet-2550 (UV-2550) dual-beam UV spectrophotometer and UV Probe software to measure the full absorption wavelength spectrum of the monosaccharide standard solution and find the maximum absorption peak.

### Determination of standard curve of monosaccharide solution

Ten monosaccharide concentration gradient solutions were prepared (10, 20, 30, 40, 50, 60, 70, 80, and 90 μg/mL). Added 1.0 mL of 6% phenol solution, then quickly added 5.0 mL of concentrated sulfuric acid, mixed well in time, and leaved at RT for 20 min. Distilled water was used as blank control. The absorbance of each concentration gradient solution was measured with a 722s visible light spectrophotometer at a wavelength of 490 nm or 480/482/483 nm. A standard curve was made with the concentration as the abscissa and the absorbance as the ordinate. In addition, in order to obtain the correction factor of other monosaccharides relative to glucose, it is necessary to ensure the consistency of other detection conditions. Therefore, the optimal detection conditions (490 nm) for glucose were selected in this study. And the ratio of the slopes of glucose to the monosaccharides is the relative correction factors for different monosaccharides relative to glucose.

### Practical application

Five representative polysaccharides, such as β-dextran, D-trehalose, fructo oligosaccharide, xylan and polygalacturonic acid, were selected for verification of correction factors. A polysaccharide solution with a concentration of about 40 μg/mL was prepared respectively, and the OD490 absorbance value of the polysaccharide solution was obtained by the phenol-sulfuric acid method, then the total sugar content was calculated.

## Results

### Moisture content analysis of monosaccharide standard

**Figure 2:**
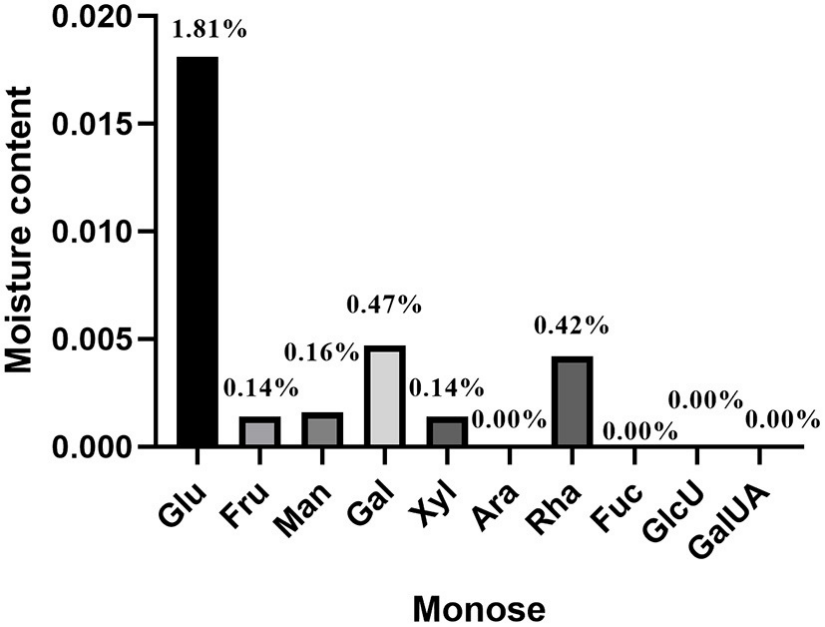
Monosaccharide water content measurement results.

Molecular configurations of different monosaccharides lead to differences in water content. In addition, RT, pressure, altitude and other factors also affect the water content of sugar molecules. The water content has a certain influence on the concentration of monosaccharide molecules. In phenol-sulfuric acid method, the concentration of the monosaccharide solution is an important measurement index. Therefore, the moisture content of 10 monosaccharides was detected. The results showed that the highest water content of glucose was 1.81%, the water content of rhamnose and galactose was about 0.40%, the water content of xylose, mannose and fructose was about 0.14%, and other monosaccharides such as fucose and arabinose and acidic monosaccharides (galacturonic acid and glucuronic acid) are relatively low in water content and can be ignored (Figure 2).

### Optimization of the phenol-sulfuric acid method

**Figure 3:**
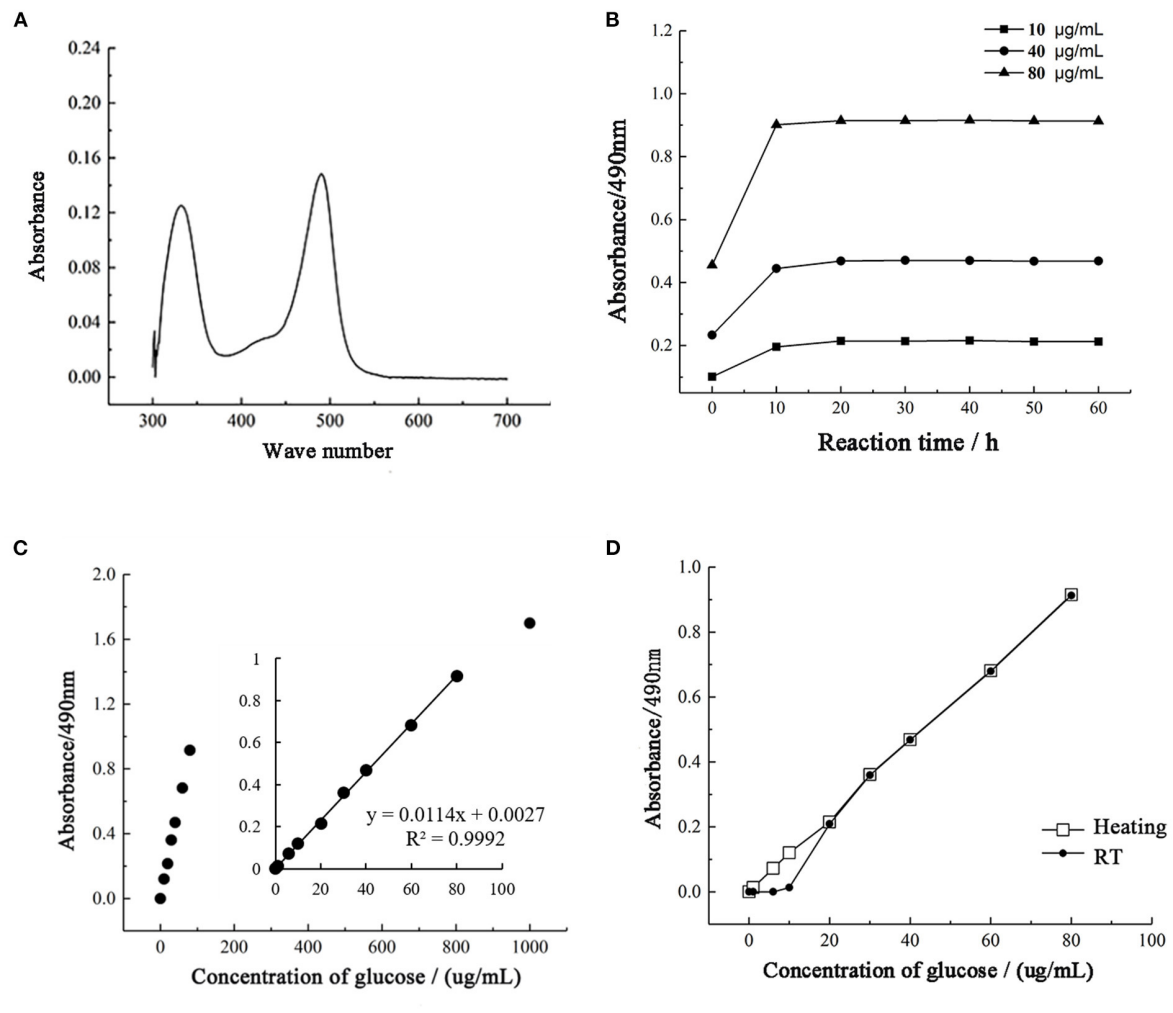
Optimization of the phenol-sulfuric acid method. **(A)** The UV-spectrum of samples. **(B)** The coloration stability of phenol sulfuric acid assay. **(C)** The range of detection for total sugar by phenol sulfuric acid assay. **(D)** The influence of heating on the phenol sulfuric acid assay.

After the phenol-sulfuric acid reaction, the glucose standard solution has two characteristic absorption peaks at 330 and 490 nm, and the absorption intensity at 490 nm is higher than 330 nm, so 490 nm is used as the detection wavelength of the phenol-sulfuric acid method (Figure 3A). During the detection process, the color stability of 10, 40, and 80 μg/mL standard grape solutions were tested. As shown in Figure 3B, the absorbance increased within 10 min after adding 6% phenol solution, reached equilibrium after 20 min, and remained stable within 1 h. The results show that the color development time of 20 min is sufficient to ensure that the absorbance of the solution to be tested reaches a stable value, and the absorbance remains stable within 1 h. At the same time, the detection results of each standard solution showed a good linear concentration relationship, which ensured the accuracy of quantitative detection.

To determine the quantitative detection range of the phenol-sulfuric acid method, a standard grape solution of 0–1,000 μg/mL was selected for testing and analysis, and the results were linearly fitted to determine the linear range. As shown in Figure 3C, the phenol-sulfuric acid method can achieve accurate and quantitative detection of total sugar in the range of 0–100 μg/mL. When the concentration exceeds 100 μg/mL, the increase in absorbance value will deviate from the range of linear quantification. Since the more accurate linear range of absorbance value is 0.2–0.8, 0–80 μg/mL standard glucose solution is selected to measure the standard curve. And the correlation coefficient *R*^2^ of the linear regression equation is 0.9992, indicating that within this range, the phenol-sulfuric acid method can accurately determine the total sugar content of the liquid to be tested.

The current study is controversial in the color development process of the phenol-sulfuric acid method. Through the study (Figure 3D), it was found that under unheated conditions, it showed a good linear trend at 10–80 μg/mL, while the absorbance value of standard glucose solution below 10 μg/mL was close to 0. Under heating conditions, both 1 and 5 μg/mL were within the linear quantitative range, indicating that heating treatment can extend the linear detection range and reduce the detection limit of the method. At the same time, in the detection range of 20–80 μg/mL, the detection results under the two conditions were highly consistent. It shows that heating does not affect the final detection result, but only reduces the detection limit of the method.

According to the experimental analysis results, the final detection wavelength of the phenol-sulfuric acid method was determined to be 490 nm, and the color development condition was static for 20 min at RT.

### Improvement of phenol-sulfuric acid method

**Figure 4:**
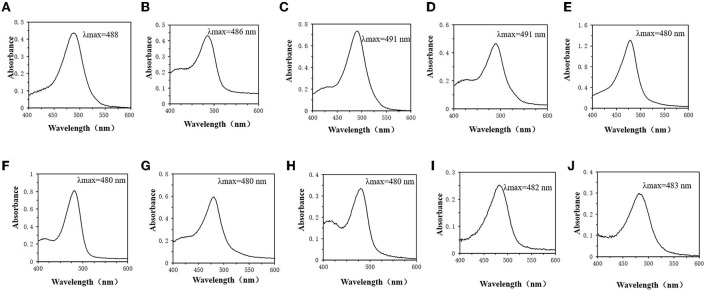
Monosaccharide UV absorption spectrum. **(A)** Glucose. **(B)** Fructose. **(C)** Mannose. **(D)** Galactose. **(E)** Rhamnose. **(F)** Xylose. **(G)** Fucose. **(H)** Arabinose. **(I)** Galactose Uronic acid. **(J)** Glucuronic acid.

By detecting the optimal absorption wavelength of the monosaccharide standard solution. It can be discovered that the spectral absorption maxima of glucose (Figure 4A), fructose (Figure 4B), mannose (Figure 4C) and galactose (Figure 4D) are around 490 nm. Rhamnose (Figure 4E) and fucose (Figure 4F), also known as 6-deoxy-L-mannose and 6-deoxy-L-galactose, have a spectral maximum absorption wavelength around 480 nm. The spectral maximum absorption wavelengths of xylose (Figure 4G) and arabinose (Figure 4H) in the pentoses are also around 480 nm. Acidic sugars, such as galacturonic acid (Figure 4I) and glucuronic acid (Figure 4J), have spectral absorption maxima around 483 nm.

**Figure 5:**
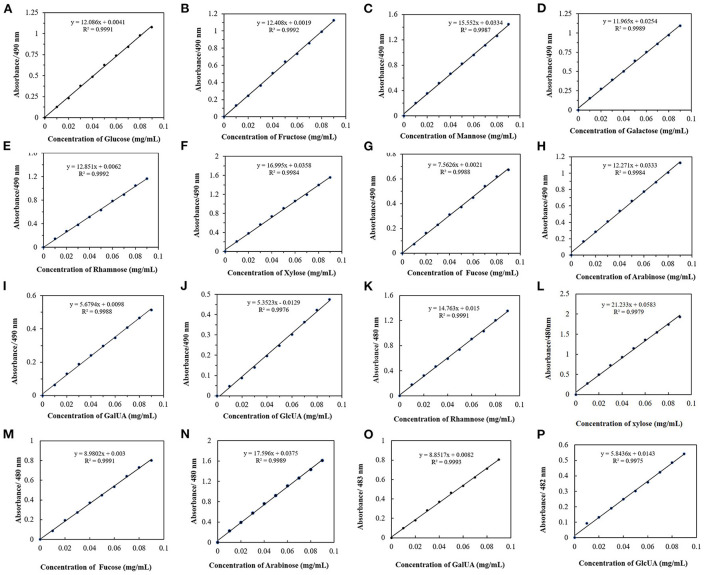
Monosaccharide UV absorption standard curves. **(A–J)** Standard curve of ten monosaccharides at 490 nm. **(K–P)** Standard curves of six monosaccharides at their maximum absorption wavelengths.

Standard curves of monosaccharides at their corresponding absorption maximum wavelengths were also obtained (Figures 5A–D, J–P). The slope represents the color rendering power of the monosaccharide in the phenol-sulfuric acid method. Through comparative analysis, it was found that when the monosaccharide was at the maximum absorption wavelength, the slope of the detected standard curve was larger. Although 490 nm is generally used as the detection wavelength of the phenol-sulfuric acid method in a large number of studies, for heteropolysaccharide samples with low concentrations, it is best to choose the maximum absorption wavelength of the sample as the detection wavelength, which is beneficial to alleviate the experimental error caused by low absorbance values. In addition, to find the rule of monosaccharide color developing ability, the standard curves of all monosaccharides at 490 nm (Figures 5A–J) were compared and analyzed. And the ratio of the slopes of glucose to the monosaccharides is the relative correction factors for different monosaccharides relative to glucose (Table 1). Since the larger the slope, the stronger the color rendering ability of the monosaccharide. The closer the relative correction factor is to 1, the smaller the difference in color rendering ability between the monosaccharide and glucose. By comparing the slopes and correction factors of monosaccharides, it can be found that the slopes of fructose, galactose, rhamnose, and arabinose are basically the same, and the relative correction factor is close to 1, while the slopes of mannose and xylose are similar, and their color rendering ability higher than glucose. The fucose and the representative of acidic sugars (galacturonic acid and glucuronic acid) were significantly lower than those of glucose. For monosaccharides with weak color rendering ability, the amount detected in the reaction system is usually low, and it is difficult to detect the accurate content in a complex solution environment. Therefore, according to this study, the total sugar content of heteropolysaccharides composed of fructose, galactose, rhamnose, and arabinose can be directly calculated using the standard curve of glucose. The heteropolysaccharides composed of xylose and mannose, fucose and acid sugar can be multiplied by the correction factor to obtain the corrected total sugar content.

**Table 1:** Regression equation and relative correction factors of glucose and other monoses.

**Monose**	**Regression equation**	***r***	**Relative correction factor**
Glu	A = 12.086C + 0.0041	0.9991	**—**
Fru	A = 12.408C + 0.0019	0.9992	0.97
Man	A = 15.552C + 0.0334	0.9987	0.78
Gal	A = 11.965C + 0.0254	0.9989	1.01
Rha	A = 12.851C + 0.0062	0.9992	0.94
Xyl	A = 16.995C + 0.0358	0.9984	0.71
Fuc	A = 7.5626C + 0.0021	0.9988	1.6
Ara	A = 12.271C + 0.0333	0.9984	0.98
GalUA	A = 5.6794C + 0.0098	0.9988	2.13
GlcUA	A = 5.3523C-0.0129	0.9976	2.26

A, absorbance; C, concentration.

### Practical application verification

To verify the accuracy of the correction factor, 5 heteropolysaccharides were selected from three types. β-dextran refers to a homopolysaccharide composed of glucose as a monosaccharide. D-trehalose is a disaccharide formed by two glucoses through α,α-1 → 1 bonds. Fructooligosaccharides are mainly composed of glucose and fructose. Xylan and polygalacturonic acid are composed of xylose and galacturonic acid, respectively. Through the detection of total sugar content, it was found that using glucose as the standard, the detected concentrations of glucan, D-trehalose and fructooligosaccharide (Table 2) were 35–37 μg/mL, which was close to 40 μg/mL, and the concentration of xylan was 57.08 μg/mL, slightly higher than the true concentration of xylan (42.6 μg/mL). while the concentration of polygalacturonic acid is about 20.8 μg/mL, which is much lower than the true concentration of polygalacturonic acid (41.6 μg/mL). After correction by the correction factor, the concentration of xylan was 40.53 μg/mL, and the concentration of polygalacturonic acid was 44.34 μg/mL, which was close to the real concentration of polysaccharide. Therefore, this experiment verifies the accuracy of the correction factor, and at the same time, the phenol-sulfuric acid color developing system can have higher accuracy through the correction.

**Table 2:** Practical application of correction factors in polysaccharides.

	**β-dextran**	**D-Trehalose**	**Fructooligosaccharides**	**Polygalacturonic acid**	**Xylan**
True concentration/(ug/mL)	37.6	38.4	39.2	41.6	42.6
Detection concentration /(ug/mL)	35.35	35.65	37.22	20.81	57.08
Relative correction factor	-	-	0.97	2.13	0.71
Corrected concentration/(ug/mL)	35.35	35.65	36.11	44.34	40.53

## Discussion

The phenol-sulfuric acid method is one of the main methods to detect total sugar content (22, 23). With the increasing number of polysaccharide species, accurate quantitative analysis by the phenol-sulfuric acid method becomes more difficult and challenging (24, 25). Numerous studies have improved the phenol-sulfuric acid method for different samples (26, 27). For example, in a sludge sample containing excess persulfate, NaSO was pre-added to prevent persulfate from affecting the determination of total sugar content by the traditional phenol-sulfuric method (28). In addition, the phenol-sulfuric acid method typically uses glucose (or a monosaccharide) as a standard to quantify the total sugar content of a sample (29, 30). However, there are significant differences in the structures and functional groups of different monosaccharides (31). For some heteropolysaccharides containing multiple monosaccharides, using glucose (or a certain monosaccharide) as the standard will lead to large errors in the detected total sugar content. Therefore, in this study, on the basis of determining the optimal detection conditions, the influence of different monosaccharide compositions on the quantitative analysis of total sugar measured by phenol-sulfuric acid method was explored, and the calibration factor of each monosaccharide relative to glucose was further obtained. In the improved phenol-sulfuric acid method, glucose can be used as the standard, combined with the monosaccharide composition of the heteropolysaccharide, and the accurate total sugar content can be obtained through the correction of the correction factor. Finally, through comparative analysis, the color rendering ability of monosaccharides was preliminarily evaluated and classified, and the monosaccharides with large deviations were corrected according to the correction factor, which provided a reference for more accurate calculation of the total sugar content of heteropolysaccharides in actual samples.

There is a close relationship between the moisture content of the sample and the concentration (32). During the measurement of total sugar content, the moisture content of the standard product was too high, which may lead to inaccurate test results. In this study, the water content of 10 common monosaccharides was determined. The water content of glucose, mannose and galactose was relatively high, and the water content of glucose was as high as 1.81% (Figure 2). Therefore, the drying and dehydration of glucose is an important step in making the standard curve of glucose. In addition, the detection conditions of the phenol-sulfuric acid method are different in different literature reports. For example, one study used heating at 100°C for 20 min during color development (25), but in another study, no heat treatment was performed (33). Therefore, in this study, the temperature, detection range, static time and other detection conditions of the phenol-sulfuric acid method were evaluated. When glucose was used as the standard, the final detection conditions of the phenol-sulfuric acid method were static for 20 min at RT for color development and detection of absorbance at 490 nm.

The difference in the color rendering ability of monosaccharides may be the main factor affecting the inaccuracy of the detection of total sugar content (34, 35). In this study, the standard solutions of 10 monosaccharides were scanned at full wavelength to determine the maximum absorption wavelengths of different monosaccharides. It was found that hexoses, such as glucose, fructose, mannose and galactose, have spectral absorption maxima around 490 nm. In addition, according to some functional groups, hexoses can be classified as aldose or ketose (36). Glucose is aldohexose, while fructose is ketohexose (37). Glucose also has two different structural forms, and the ring-shaped fructose also has two different structures, alpha and beta. Due to the similar structure of the hexose (38), the reactions produced in the phenol-sulfuric acid method are relatively consistent. There is no significant difference in the color rendering power, which may be the reason that the measured spectral range and absorbance are almost the same. Rhamnose, fucose, and pentose (xylose and arabinose) have maximum absorption wavelengths around 480 nm. Rhamnose and fucose, also known as 6-deoxy-L-mannose and 6-deoxy-L-galactose, respectively (39, 40). It can be found that the wavelength of maximum absorption changes as the hydroxyl groups of mannose and galactose are replaced by hydrogen (Figure 4). While it may be due to the low solubility of pentose in ethanol (41), and the low concentration of pentose leads to a lower optimal wavelength of absorbance and UV absorption in the phenol-sulfuric acid color developing system. In addition, acidic sugars such as galacturonic acid and glucuronic acid have spectral absorption maxima around 483 nm. Therefore, for heteropolysaccharides with different monosaccharide compositions, the maximum absorption wavelength may be in the range of 480–490 nm.

To obtain a more accurate total sugar content by using the phenol-sulfuric acid method, 10 different monosaccharides were used to create a standard curve in this study. The other monosaccharides were corrected with glucose as the standard to obtain the correction factor. By comparative analysis of correction factors, the 10 monosaccharides were divided into 3 categories according to their color rendering ability. And selected representative heteropolysaccharides in three categories to verify the accuracy of the correction factor. It is clear that the total sugar content of heteropolysaccharides composed of fructose, galactose, rhamnose, and arabinose can be directly calculated using the standard curve of glucose. While the heteropolysaccharides composed of xylose and mannose, fucose and acid sugar can be multiplied by the correction factor to obtain the corrected total sugar content.

The improved phenol-sulfuric acid method not only did not increase the complexity of the experiment, but also made the detection more accurate, especially significantly improved the accuracy of the total sugar content of acidic heteropolysaccharides. However, since this method ignores the influence of different proportions of monosaccharides on the quantitative analysis of total sugar content, the detection accuracy of heteropolysaccharides composed of different types of monosaccharides may be lower than that of heteropolysaccharides composed of the same type of monosaccharides. Therefore, in future studies, the experimental scheme can be further optimized by discussing the proportion of different types of monosaccharides in the heteropolysaccharide, which improves the rigor and accuracy of the quantitative analysis of total sugars.

## Conclusion

Based on the traditional phenol-sulfuric acid method for the determination of total sugar content in substances, this study revealed the influence of the sample moisture content, heating conditions, reaction time, and optimal absorption wavelength on the stability and accuracy of the phenol-sulfuric acid method. In addition, using 10 monosaccharides as target detection substances, a phenol-sulfuric acid chromogenic system for the determination of total sugar content in heteropolysaccharides was constructed. And the other monosaccharides were corrected with glucose as the standard, and the correction factors were obtained. The 10 monosaccharides were preliminarily divided into 3 categories according to the correction factor. Then, the known concentration of heteropolysaccharide was selected for verification, and it was preliminarily proved that the correction of the relative correction factor could obtain a more accurate total sugar content. This study not only significantly improves the accuracy of the phenol-sulfuric acid method, but also classifies the color rendering abilities of different monosaccharides, further simplifying the experiment, which has better application potential in the detection of total sugar content in actual complex samples.

## Data availability statement

The original contributions presented in the study are included in the article/supplementary material, further inquiries can be directed to the corresponding authors.

## Author contributions

FY, XL, and ML: conceptualization and methodology. FY: investigation and writing. JZ, JX, and TN: data curation and review. XL and ML: funding acquisition. All authors contributed to the article and approved the submitted version.

## Funding

This work was financially supported by Chinese Universities Scientific Fund (Grant No. 2452022025), and Key point Research and Invention Program of Shannxi Province (Grant No. 2021ZDLNY05-06).

## Conflict of interest

The authors declare that the research was conducted in the absence of any commercial or financial relationships that could be construed as a potential conflict of interest.

## Publisher's note

All claims expressed in this article are solely those of the authors and do not necessarily represent those of their affiliated organizations, or those of the publisher, the editors and the reviewers. Any product that may be evaluated in this article, or claim that may be made by its manufacturer, is not guaranteed or endorsed by the publisher.
